# Causal reasoning in rats' behaviour systems

**DOI:** 10.1098/rsos.171448

**Published:** 2018-07-04

**Authors:** Robert Ian Bowers, William Timberlake

**Affiliations:** 1 Cognitive Science, Indiana University, Bloomington, IN, USA; 2 Psychological and Brain Sciences, Indiana University, Bloomington, IN, USA; 3 School of Psychology, Universidade do Minho, Braga, Portugal

**Keywords:** causal reasoning, behaviour systems, Bayesian networks, causal model theory, cognitive modelling, *Rattus norvegicus*

## Abstract

Conceiving of stimuli and responses as causes and effects, and assuming that rats acquire representational models of causal relations from Pavlovian procedures, previous work by causal model theory proponents attempted to train rat subjects to represent stimulus A as a cause of both stimulus B and food. By these assumptions, with formal help from Bayesian networks, self-production of stimulus B should reduce expectation of alternative causes, including stimulus A, and their effects, including food. Reduced feeder-directed responding to stimulus B when self-produced has been taken as evidence for a general causal reasoning capacity among rats involving mental maps of causal relations. Critics have rejoined that response competition can explain these effects. The present research replicates the key effect, but uses continuous and finer-grained measurement of a broader range of behaviours. Behaviours not recorded in previous studies contradict both prior explanations. Even results cited in support of these explanations, when measured in finer detail and continuously over longer periods, show patterns not expected by either view, but supportive of a specific-process approach with attention to motivational factors. Still, the abstract prediction from Bayesian networks holds, providing a potentially complementary normative analysis. Behaviour systems theory provides firmer framing for such theories than representational-map alternatives.

## 1. Introduction

Amid oceans of research and theory on learning—sometimes conceived in cause–effect terms, sometimes not—questions about post-learning processing, of the manner theories of causal reasoning concern, have received comparatively little research attention. In one such study, Blaisdell *et al*. [1] (‘Causal reasoning in rats’) showed that behaviour of rats conformed to a prediction of a theory of causal reasoning not anticipated by alternatives.

**Figure 1.**
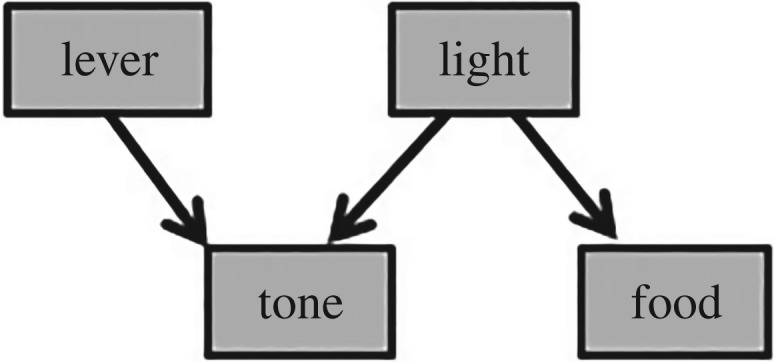
The causal model hypothesized to have been acquired in the experiment. Arrows indicate causal relations: light causes tone; light causes food; lever causes tone (see footnote 1).

The rationale of the experiment involves two very different theories of causal reasoning. Following causal model theory (CMT) [2], Blaisdell *et al*. [1] assumed that rats would perceive and represent preceding stimuli in a Pavlovian setting as causes of following stimuli, and integrate multiple Pavlovian associations into representational models of causal relations. With these assumptions, they devised a training procedure to produce in rats the representational model illustrated in figure 1. In a final test, the rats found a lever in the chamber. For one group of subjects, contacting the lever produced the familiar tone stimulus from the training trials. These subjects were assumed to quickly come to see the tone as an effect of lever press. The other group received the same pattern of stimulus presentation irrespective of their actions (yoked control). Table 1 depicts the design.

**Table 1.** Procedure and Blaisdell *et al*.'s [1] prediction.

group	phase 1	phase 2	test	prediction
RI (response independent)	light, then tone	light, then food	tone (yoked to LD)	default response levels
LD (lever dependent)	light, then tone	light, then food	lever produces tone	reduced response levels

If the model in figure 1 is, in some sense, an accurate depiction of what Blaisdell *et al*.'s [1] subjects acquired, a second theory makes a prediction. Bayesian networks is foremost a smart way to deal with conditional probabilities, and this makes it available as a normative theory of what a rational agent ought to expect under a range of conditions [3]. Bayesian networks does not predict the model in figure 1, but it does predict what a rational possessor of such a model might expect when presented information about the modelled events. The path from lever to food involves two conditional dependencies. First, two effects (tone and food) of a common cause (light) become dependent on each other when the occurrence of the light is unknown [3]. Hearing the tone, expectation of food should heighten. That rats check food troughs in such circumstances is neither news (cf. [4]), nor problematic for mainstream views of learning.

Second, two causes (lever contact and light) of a common effect (tone) become dependent on each other, given the occurrence of tone [3]. In this case, the conditional relationship is negative; hearing the tone, having contacted the lever reduces otherwise heightened expectation of light. This in turn reduces expectation of other effects of the light, such as food delivery. By analogy, having pressed one's own doorbell, the chime is no longer good evidence of a caller. By this reasoning, rats should reduce responding to a stimulus indirectly predictive of food when that stimulus can be attributed to an alternative cause unrelated to food, such as one's own lever press. This is the novel prediction Blaisdell *et al*. [1] supported: rats checked a food trough less in response to a lever-produced tone than did yoked control subjects to a response-independent (RI) tone. They concluded that this was evidence for ‘the core competency of reasoning with causal models' (p. 1022).

This is the starting point of the current study: the prediction in focus has been supported. Three potential explanations are examined: (i) causal model theory, the view given by the authors of the original study [2,5]; (ii) response competition [6]; and here we present (iii) a behaviour systems-derived hypothesis that the divergence of responding between the two groups of rats is due to their being in different motivational circumstances. Although all three explanations account for the key result described above, they differ in the specific response topographies expected to produce it. To differentiate among these explanations, the current study replicates the demonstration using a finer-grained measurement scheme, recording continuously, and recording other pertinent behaviours. Cognitive modelling techniques [7] are applied to address some ramifications of each hypothesis.

### 1.1. Three explanations

#### 1.1.1. Causal model theory

Blaisdell *et al*. [1] presented their results as suggesting a general capacity for abstract causal reasoning, speaking in terms of causal models, and elsewhere, as supporting CMT [2,5]. By this view, ‘animals have a natural tendency to translate covariations into causal-model representations’ [2, p. 310]. The animal will encode relations according to mental maps of causes and effects that then provide a basis for reasoning about those relations. CMT attributes to the animal a kind of representational theory or model consisting of structured representations of causal relations. By CMT, figure 1 is not merely a computational-level abstraction (*sensu* [8]), but a literal depiction of a representational map acquired by the subjects.^1^ The reduction in response to the self-produced tone is viewed as the animal's mentally discounting the stimulus as an indicator of food.

#### 1.1.2. Response competition

Dwyer *et al*. [6] replicated the primary demonstration in focus in [1], but measured lever pressing in addition to nosing of the feeder, and found group differences in both measures. Unsurprisingly, lever pressing was much greater on lever press-produced presentations. Dwyer *et al*. [6] concluded that the animal might nose the feeder less during lever press-produced presentations simply due to being otherwise occupied at the onset of the tone with an incompatible response, pressing the lever. Importantly, by this explanation, the results of [1] are an artefact of the specific procedure, rather than reflecting a quality of the animal, and so appeal to a normative theory of causal reasoning is not appropriate, and the apparent correspondence with Bayesian networks is spurious.

#### 1.1.3. Behaviour systems

Behaviour systems perspectives [10–14] (overviewed in [15]) assume that behaviour exists in systems with identifiable structure, and place focus on the production, timing, and motivational context of specific sequences of behaviour. This perspective provides a third explanation for the effect in focus, that it is produced by the interdependence of feeding-related motivational states.

To understand the behaviour of rats in these circumstances requires recognition that manipulating a prey-sized protrusion, such as the lever, in a food-conditioning experiment is often an expression of feeding motivation [16]. It readily develops among rats under restricted feeding that are placed, at mealtime, in a feeding context, and resembles predatory responding of rodents to live crickets [17]. By a behaviour systems view, therefore, lever manipulation is no arbitrary operant, particularly in feeding contexts. Likewise, nosing any available crevice is a feeding-related behaviour that readily develops in such experiments. Response levers and food troughs in standard conditioning chambers take the form they do, because they have undergone a selection process to produce reliable behaviour [13]. As part of a single system, these two behaviours occur in sequences, such that the performance of one predicts the performance of the other.

In Blaisdell *et al*. [1], behaviour was measured in two very particular circumstances that differ in an important respect. In one group, presentation begins when the animal makes lever contact. In the other, the stimulus appears irrespective of current activity or state. By a behaviour systems perspective, responding is expected to differ under these two circumstances due to differences in the current motivational state of the animal. By grabbing or biting the lever, the rat in one group is indicating a momentary high of feeding motivation. It is because the stimulus occurs at this point in the sequence of behaviour that the topography of response is so specific, and qualitatively different from when the same tone is presented at other times. As Dwyer *et al*. [6] demonstrated, rats behave differently under these two circumstances, with persistent lever contact in the former case, and primarily nosing of the feeder in the latter. Contrary to Dwyer *et al*. [6], we attribute the divergence in form of response to qualities of the animal, rather than to the circumstance. Hence, application of normative theories of causal reasoning may yet be appropriate, in principle; it is as meaningful to ask whether motivational structures follow prescriptions of Bayesian networks, as whether mental models do. Contrary to Blaisdell *et al*. [1], by whose view subjects should discount self-produced stimuli, we expect subjects to be at a motivational peak when they press.

### 1.2. A difference in measurement

The measurement scheme used in [1] and replication attempts (e.g. [6]) is insufficient for the purpose of comparing the models under consideration in three important ways: it (i) overlooks relevant behaviours, (ii) ignores relevant periods, and (iii) measures in overly gross increments. We measure multiple relevant behaviours, continuously over the entire session, and in sufficiently fine increments to address the hypotheses.

Blaisdell *et al*. [1] began recording at the onset of stimulus presentation, when the lever-dependent (LD) rat contacted the lever and the tone was presented to both animals, leaving pre-stimulus behaviour undocumented. Instead, Blaisdell *et al*. [1] compared stimulus period responding with a post-stimulus period (10–20 s after cessation). This is non-standard, and a less satisfying comparison, since effects of the manipulation may continue to affect the animals' behaviour differentially, and it provides no way of determining whether the subjects were in similar condition at the moment the stimulus was presented. In this case in particular, since presentation was response-dependent for one group and response-independent for the other, the pre-stimulus period has specific relevance. In the current study, recording of behaviour is continuous, which allows recovery of the crucial time period immediately prior to lever contact.

Blaisdell *et al*. [1] (also [6,18]) measured behaviour in 10 s increments. Their causal model hypothesis did not motivate finer-grained measurement. However, to satisfactorily address response competition does require temporally narrower focus. The levers in Blaisdell's and Dwyer's chambers are next to the food trough. Rats can and do engage both simultaneously, keeping one hand on the feeder while working the lever. If having contacted the lever impedes subsequent behaviour tangibly, the interference should be disproportionately early in the stimulus period, which cannot be determined from data binned in 10 s intervals. It is a time-sensitive hypothesis, and to test it requires consideration of behaviour in finer increments. The behaviour systems-based hypothesis is similarly time-sensitive. The present study measures behaviour in 2 s periods.

Blaisdell *et al*. [1] measured only one response: nosing of the feeder. Dwyer *et al*. [6] also measured lever contact, ostensibly to assess the role of response competition. By a behaviour systems view, three categories of related behaviours are pertinent to measure in a feeding situation. In addition to nosing of the feeder (a focal-appetitive behaviour), and lever contact (a consummatory behaviour), we measured a general-appetitive behaviour, movement around the chamber. Although these measures are motivated by a behaviour systems perspective, and the other two views do not explicitly anticipate patterns of these other behaviours, this does not imply theoretical neutrality. Some patterns of these other behaviours are inconsistent with the other two views, and so these measures help to differentiate among the available hypotheses.

## 2. Methods

In addition to the differences in measurement stressed above, our experiment uses a different apparatus, different strain and sex of rat, and different food than in [1] (and [5], which was faithful to the former).

### 2.1. Subjects

Twenty experimentally naive female Sprague–Dawley rats served as subjects (10 per group). For the course of the experiment, they were housed individually on a 12-hour light:dark schedule. They were fed once daily, 1–3 h after dark, and maintained at a minimum of 85% of their free-feeding weights (median weight: 290.5 g; median age: 499 days). Water was freely available in home cages.

### 2.2. Apparatus

The testing apparatus consisted of pairs of chambers made of sheet metal (50 × 35.5 × 30 cm), Plexiglas ceiling, and accessed by a hinged Plexiglas front wall. These were in sound attenuating cabinets in one room, 20 cm apart, and linked to data-logging and control equipment in an adjacent room. The food trough (3.1 cm wide, 5.9 cm high, 1.8 cm deep, 4 cm above floor) detected intrusions by an infrared sensor. Food delivery consisted of two 45 mg Testdiet purified pellets (1 s apart) released into the feeder. The lever (3.6 cm wide) was retractable, and only extended in phase 3 (5.2 cm from the feeder; 4 cm above the floor, 5.1 cm from the back wall). The light and speaker were next to each other 1.6 cm above the lever. The speaker emitted an 82 dB white noise. The light stimulus was a flashing green 28 V jewel light (1 s^−1^). Two pressure-sensitive platforms in the floor of the chambers covered most of the back wall (10.2 cm wide).

### 2.3. Procedure

Except where noted, procedures were similar to those in [1] (experiment 2a). All phases of the experiment were conducted in the dark portion of the day, and in dark experimental chambers (in [1], chambers were lit). Subjects were run in pairs.

#### 2.3.1. Exposure to the apparatus

For 2 days, subjects were placed in the conditioning chambers for 30 min, with 10 food pellets. All subjects ate the pellets by the second day.

#### 2.3.2. Phase 1

On each of 4 days, subjects were presented with six presentations (i.e. 24 total) of a 10 s flashing (‘light’), followed immediately by a 10 s white noise stimulus (‘tone’). Presentations were 3–7 min apart (mean interval, 5 min). These sessions were 35 min long. In [1], there was a second auditory stimulus presented alone not included here.

#### 2.3.3. Phase 2

On each of 2 days, 12 presentations of the 10 s flashing light were followed immediately by food delivery (3–7 min apart, mean: 5 min, total duration: 62 min).

#### 2.3.4. Phase 3

Levers were extended for a single 30 min test period (in [1], these were 60 min). Whenever the LD subject contacted the lever, the 10 s tone sounded in its own chamber, and in a yoked chamber occupied by its RI partner. A 30 s refractory period (10 s in [1]) assured spacing of stimuli. RI subjects could not produce the stimulus.

### 2.4. Analyses

The conditioning chambers automatically recorded whether, in each 0.1 s interval, the subject was in contact with the lever, in the food trough, or on either of two platforms along the back chamber wall. This yields a value of 0–20 for each dependent variable, for each 2 s period. Ten-second bins used in quantitative analyses are the sum of five adjacent 2 s bins.

#### 2.4.1. Quantitative comparisons

Repeated-measures analyses of variance were conducted to compare groups on each of the three dependent variables over test sessions (phase 3) for 10 s periods immediately before and during stimulus presentation. Adjacent pairs of observations were combined in blocks of two. Similar analyses were conducted on baseline measurements. These consisted of all 10 s periods throughout the 30 min test session not overlapping with a 30 s stimulus period. A baseline value was ascertained for each 3 min block, yielding 10 values for each measure and subject. Analyses assume first-order ante-dependent covariance structure [19] on account of unequal numbers of observations among subjects, unequal spacing of observations, and a serial quality to the within-subject correlations, expected due to extinction over non-reinforced presentations. Informative *post hoc* tests are described where relevant.

#### 2.4.2. Qualitative modelling

The modelled data are the aggregate feeder-directed response quantities for each 2 s parcel over the 30 s period of interest surrounding stimulus presentations (i.e. starting 10 s prior to presentation). The mean of the remainder (i.e. excluding the same 30 s periods) of 2 s periods throughout sessions for all subjects provide a baseline. The Nelder–Mead simplex technique [20] was used to estimate unspecified parameter values, using sum of squared errors of prediction (SSE) between model and data as a measure of fit. Note that parameter values are specific to the particular variables and measurement scheme used, including capacity for response, time course, etc. Since the models considered vary in complexity, but are not nested, Akaike information criterion (AIC) was used to quantify fit. A model that fits in form may still underestimate or overestimate the extent of an effect. Therefore, in addition to assessing models with optimally fitting parameters, each model was also assessed with parameter values that would be required to produce the extent of difference observed.

## 3. The models

The three views described are all consistent with the finding [1] that rats nose a food trough less on lever-dependent presentations (LD condition) than response-independent presentations (RI condition), and they agree regarding response patterns expected on RI presentations. What differentiates the three interpretations is the behaviour of LD subjects.

While the behaviour systems-based hypothesis predicts distinct response topographies in the two conditions, the predictions of both causal model and response competition hypotheses concern a reduction of feeder-directed responding in the LD condition relative to the RI condition. Neither view predicts specific magnitudes of this difference. However, since they attribute different factors for the reduction, different patterns of behaviour are implied. Thus, for these two views, we begin by establishing a descriptive model of RI responding to provide a basis upon which to apply these differing models of reduction.

We can model the RI condition responding as a Gaussian function as follows:

3.1 $$f(x)=h{\text{e}}^{-({\left(x-p\right)}^{2}/2k^{2})}.$$

The term *h* specifies the height of the curve. The position of the peak is specified by *p*, which corresponds to time (in 2 s periods of measurement, 1–15, for a 30 s period, beginning 10 s prior to the tone, including the 10 s stimulus period, and a 10 s post-stimulus period). Kurtosis is specified by *k*. A Gaussian function yields an interpretable response curve, with an initial gradual rise from a baseline, and a similarly gradual return to the same baseline. As the model of RI condition responding, we take the best fitting function of this form; its parameters specifying height, position and kurtosis are, respectively, *o*_h_, *o*_p_, *o*_k_ (constants):

3.2 $$\text{RI}(x)=o_{h}{\text{e}}^{-({\left(x-o_{p}\right)}^{2}/2{o_{k}}^{2})}.$$

### 3.1. Expectation reduction hypothesis

By CMT, the lower response to the self-produced tone (in [1,6]) is due to reduced expectation. Thus, a curve that describes responding well on RI presentations should, by CMT, similarly provide a good model of responding on LD presentations, except that the height of the curve will be lower (i.e. *h* is unspecified but lower and non-negative, 0 ≤ *h* < *o*_h_), as in equation (3.3):

3.3 $$\text{L}{\text{D}}_{\mathrm{ERH}}(x)=h{\text{e}}^{-({\left(x-o_{p}\right)}^{2}/2{o_{k}}^{2})}.$$

As this model is essentially about a reduction of expectation, it applies similarly to any explanation that views the Blaisdell *et al*. [1] demonstration in such terms. For instance, the associative model of Kutlu & Schmajuk [21] explains the effect by assuming an inhibitory association between the lever press and food. The prediction is the same, a general reduction of the height of the response curve. Also, views that attribute the reduction of responding following lever press to competition for processing demands in a system with limited capacity [22] might be modelled similarly. Thus, the success or failure of this model will have implications beyond CMT.

### 3.2. Response competition hypothesis

By the response competition hypothesis (RCH), the lower feeder-directed responding on lever-produced presentations is due to the animal's occupation with the lever at the beginning of the presentation. Like CMT, RCH does not predict specific response curves, but rather, a reduction in group LD responding relative to group RI. By RCH, expectation of food is similar in both conditions, and motivation is similar; *h* = *o*_h_. What is predicted to differ is latency, the position of the response curve. Therefore, the response competition model of LD condition responding we propose is again based on the descriptive model of RI condition responding, but with the position term unspecified, as in equation (3.4), with the constraint that *p* is later (*p* > *o*_p_):

3.4 $$\text{L}{\text{D}}_{\mathrm{RCH}1}(x)=o_{h}{\text{e}}^{-({\left(x-p\right)}^{2}/2{o_{k}}^{2})}.$$

A second way to model the effect of response competition is to subtract the hypothesized interference from the RI default model. As there may be a peak of maximal interference that builds and lessens with time, it is again convenient to model the interference as a Gaussian function positioned at the moment of lever contact (i.e. *p* = 6), with unspecified height (*h* > 0) and breadth (*k* > 0):

3.5 $$\text{interfere}(x)=h{\text{e}}^{-({\left(x-6\right)}^{2}/2k^{2})}.$$

Therefore, we propose equation (3.6) as a second response competition model:

3.6 $$\text{L}{\text{D}}_{\mathrm{RCH}2}(x)=\text{RI}(x)-\text{interfere}(x).$$

The same reasoning applies equally to other behaviours: if lever contact interferes with nosing the feeder, it should interfere no less and in a similar fashion with other potentially competing responses. Thus, a similar strategy can be taken with other behaviours that negatively correlate with lever contact. If only some behaviours show the negative relationship predicted, this will require explanation beyond response competition. Note that the RCH does not make predictions regarding patterns of lever contact. Occurrence of lever contact in group LD is given by the procedure; persistence of lever contact is not a prediction.

### 3.3. Behaviour systems-based behavioural sequence model

By a behaviour systems view, RI and LD subjects encounter the tone under qualitatively different motivational circumstances. If lever contact is an expression of focused feeding motivation, by grabbing the lever, the LD animal is showing evidence of being at a motivational climax when the tone appears and behaviour is measured. This contrasts with the alternative views considered, by which responding among LD animals is reduced generally. The contrast is especially sharp with views, such as CMT, that attribute the group difference to reduced expectation among LD subjects. Since the difference is hypothesized to be qualitative, unlike the above models, our behavioural sequence model of LD responding cannot bootstrap on a descriptive model of the RI data. However, there are direct theoretical grounds for predicting specific response topographies, particularly for the case of the LD subject, whose current motivational condition is revealed by its behaviour.

**Figure 2.**
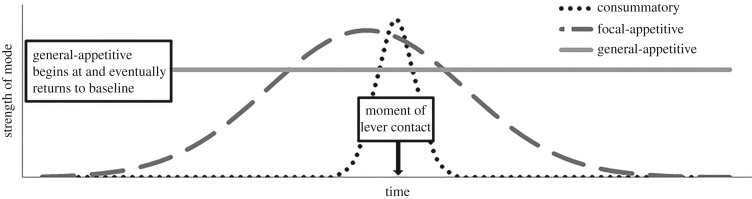
A behaviour systems model of the motivational changes accompanying sequences of feeding behaviour (cf. [24–26]).

The form of the response, by this view, is complex, involving general-appetitive, focal-appetitive and consummatory responses (cf. [23]), each with specific relations to the others, as depicted in figure 2 (after [24–26]). In the conditioning chambers, the recessed food trough affords nosing, and the protruding lever affords grabbing and biting. A rat in a consummatory motivational state related to feeding will tend to grab and bite prey-sized protrusions [17] like the lever;^2^ a rat in focal-appetitive mode will tend to nose the food trough, where food has been found before. Conveniently, these are the two responses in focus. General-appetitive is visible as movement away from the food trough, which in the restricted space of typical conditioning chambers resembles pacing, much like a polar bear in a zoo circles its enclosure in the hours prior to mealtime [28].

In the response-independent condition, the subject receives the tone irrespective of behaviour or state. As consummatory moments are brief [29], it should more often encounter the tone under focal-appetitive motivation and nose the feeder, which can be modelled as Gaussian. Hence, all three views are content with the same model for the RI condition.

When the LD subject produces the stimulus, it is already in the throes of a feeding behaviour sequence. Lever contact does not appear in a behavioural vacuum. As a consummatory response, it is short-lived, such that the timing of spontaneous lever contact can be placed in a relatively narrow window, the dotted curve in figure 2. By controlling the presentations to appear in this very specific and reliable moment in the motivational sequence, when the rat is furthermore in a specific position and orientation with respect to the lever, a circumstance is isolated in which the model predicts response topographies with especial precision.

Although the progression of feeding-related motivational states is assumed to be continuous, as diagrammed in figure 2, the behaviours produced are discrete and sequential. Feeding behaviours occur together, but in sequences, rather than all at once, leading the animal along a chain of behaviour patterns that progressively narrow upon acquiring prey. A rat engaged in consummatory responding towards the lever is temporarily less inclined to nose the feeder. The model therefore involves the interaction of two complementary motivational progressions: focal-appetitive mode (‘focal’), encouraging feeder-directed responding, and consummatory mode (‘consum’), producing consummatory behaviours, to the exclusion of feeder-directed responding. Thus, our behavioural sequence model of LD condition feeder-directed responding is as in equation (3.7):

3.7 $$\text{L}{\text{D}}_{\mathrm{BS}}(x)=\mathrm{focal}(x)-\mathrm{consum}(x).$$

That is, nosing of the feeder is predicted by the model of focal-appetitive mode, minus the model for consummatory mode. Again, these two progressions can be appropriately modelled as Gaussian functions, although this is a simplification of the original Silva–Timberlake [24–26] model, in which no commitments were intended to symmetrical functions.

As the timing of lever contact relative to stimulus presentation is given by the procedure, the position of the consummatory mode model is specified: the moment of lever contact, *p* = 6. Breadth and height are free parameters. These are the same constraints as in the interference component of RCH2, accentuating the importance of how these models treat the positive component differently.

3.8 $$\text{consum}(x)=h{\text{e}}^{-({\left(x-6\right)}^{2}/2k^{2})}.$$

Focal-appetitive is modelled as a Gaussian (as in formula (3.1)) with all three parameters (height, kurtosis, position) unspecified, leaving five free parameters in the model.

Since the experiment focuses on a moment of peak consummatory motivation, predictions regarding lever contact are especially clear. As shown in Dwyer *et al*. [6], lever contact during stimulus periods should be greater for group LD than group RI. However, since presentations are dependent on lever contact of group LD, it is necessarily absent in pre-stimulus and baseline periods, making group comparisons of these periods trivial.

## 4. Results

### 4.1. Quantitative comparisons

#### 4.1.1. Focal-appetitive

**Figure 3.**
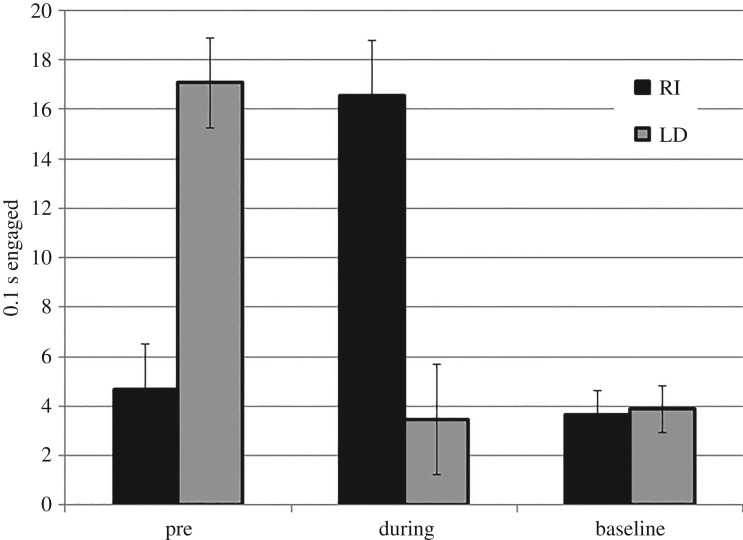
Nosing of the food trough in 10 s bins, prior to and during CS presentation, and on baseline measurements (estimated means). Error bars show estimated s.e.m.

The demonstration of primary interest in [1] was replicated: nosing of the food trough during stimulus presentation was less for group LD than group RI (*F*_1, 8.38_ = 17.32, *p* = 0.003; mean difference = 13.10, 95% CIs = 5.90–20.31; figure 3). No group difference was observed in baseline measurements (*F*_1, 31.75_ = 0.03). Both results are consistent with all three views. However, responding in the pre-stimulus period was greater for group LD than group RI (*F*_1, 5.94_ = 23.16, *p* = 0.003; mean difference = 12.40, 95% CIs = 6.08–18.71). This was predicted by the behavioural sequence model, but not by the alternatives.

#### 4.1.2. Consummation

Lever contact during stimulus presentation was greater for group LD than group RI (*F*_1, 19.59_ = 14.95, *p* = 0.001; mean difference = 11.39, 95% CIs = 5.24–17.55), as also observed in Dwyer *et al*. [6]. Given that LD subjects had just contacted the lever, this result is not surprising. Neither the causal model view nor the response competition view make predictions regarding patterns of lever contact. Such a comparison is not appropriate for the baseline or pre-stimulus periods, since timing of the tone is dependent on lever contact in one group; trivially, lever contact will be greater for RI subjects at these times. Subjects produced two to nine stimulus presentations by lever contact during phase 3 (median: 5).

#### 4.1.3. General-appetitive

No statistically reliable group differences were revealed by planned tests in movement around the chamber, as measured by presence on the two platforms along the back wall (pre-stimulus: *F*_1,10.91_ = 2.73; during: *F*_1,20.28_ = 1.33; baseline: *F*_1,46.60_ = 0.16; *p*'s > 0.1). Though not significantly greater, mean far platform values during the tone were at least as high for LD (raw mean: 26.83) as RI subjects (10.83). This is awkward for the response competition explanation: if having contacted the lever is no impediment to being on the far side of the chamber, the same starting point can scarcely be used to explain, over the same period, reduced presence at the feeder, only 4 inches away. This result is likewise counter to the causal model explanation. If reduction of expectation of food is what causes lower feeder-directed response to the tone among LD subjects, other feeding-related behaviours should be similarly reduced. Instead, both lever contact and general locomotion are at least as great in these conditions. In a related study more directly concerned with intervention [9], greater general locomotion was shown among subjects for whom a conditional stimulus was self-produced by lever contact, relative to a RI condition.

### 4.2. Model comparison

#### 4.2.1. Response-independent condition responding

**Figure 4.**
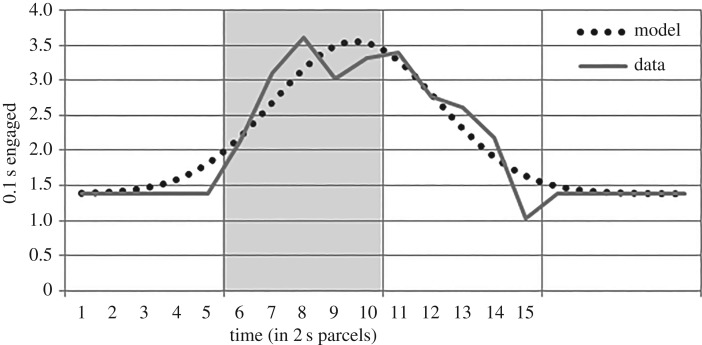
RI condition feeder-directed responding and the descriptive model. Vertical lines denote 10 s periods (tone presentation is during time parcels 6–10).

Two of the hypotheses phrase LD condition responding relative to RI condition responding. A Gaussian function (height = 2.18; position = 9.63; kurtosis = 2.59) fits the RI data well (SSE: 1.42; figure 4). This provides a default model of conditional feeder-directed responding for the specific circumstance. Consistent, baseline-level responding was assumed prior to and following presentations (time parcels 1–5 and 16–20). Baseline (1.38) was similar between groups (1.37 for group RI; 1.39 for LD).

#### 4.2.2. Expectation reduction model

**Figure 5.**
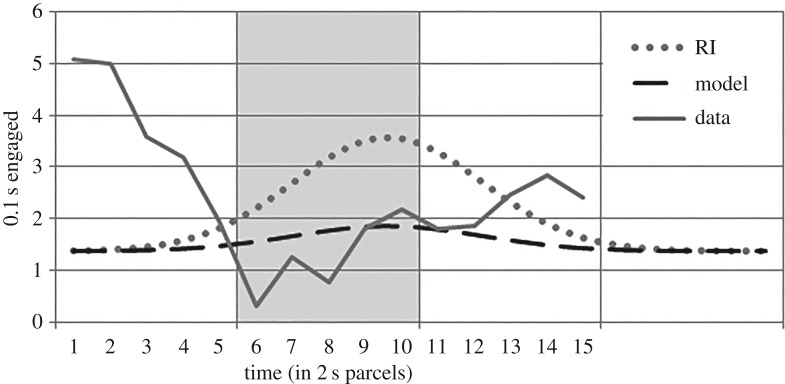
The model based on expectation reduction (*h* = 0.49), motivated by CMT. The observed LD condition feeder-directed responding and the descriptive model of RI condition responding are presented for comparison. Vertical lines denote 10 s periods. Shading indicates the stimulus period (6–10).

The height of the best fitting expectation reduction model is 0.49 (figure 5). The fit to the data is poor (SSE: 41.30; AIC: 16.68). Most obviously, it fails to predict the pre-stimulus peak in responding. No parameter settings will account for this character of the data, and this is appropriate since the hypothesis gives no reason to expect it. Secondly, this model overestimates LD response levels during the tone. Observed levels are reached only by negative parameter values (*h* = −0.16; SSE: 43.25), indicating below baseline responding, not anticipated by any view that attributes the group difference only to reduced expectation.

#### 4.2.3. Response competition

**Figure 6.**
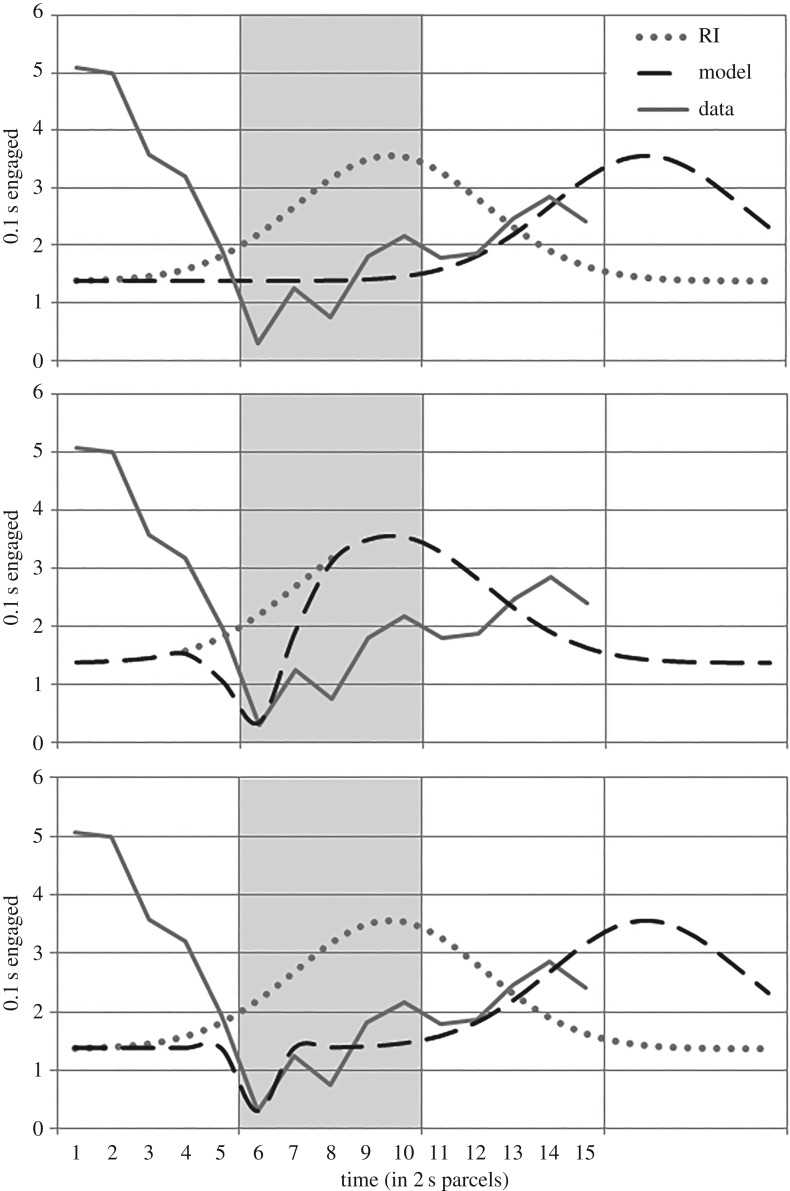
Three response competition models. The observed LD condition feeder-directed responding and the descriptive model of RI condition responding are presented for comparison. Vertical lines denote 10 s periods. Shading indicates the stimulus period (6–10). Upper panel: the delay-based model, RCH1 (*p* = 16.64). Middle panel: the subtraction-based model, RCH2 (*h*2 = 1.84; *k*2 = 0.75). Lower panel: RCH3, a combination of the delay of RCH1 with the subtraction of RCH2 (*p* = 16.64; *h*2 = 1.08; *k*2 = 0.13).

Two response competition models were proposed. The first, RCH1, models the reduction in response as a temporal shift. The best fit was found to occur at *p* = 16.64 (figure 6, upper panel). This not only fits the data poorly (SSE: 38.16; AIC: 15.89), but also attributes an infeasible delay (14 s). Contacting a lever within arm's length is not expected to delay a rat 14 s. Furthermore, regardless of parameter values, this model necessarily overestimates responding during the tone in condition LD, which was below baseline, and underestimates responding in the pre-stimulus period.

The second response competition model (RCH2) considered treats interference as a second Gaussian function to be subtracted from the default model. The best fitting parameters also fit intuitions about the extent of interference feasible, yielding an interference curve with height, *h*2 = 1.84, and breadth, *k*2 = 0.75 (figure 6, middle panel). The model follows the dip observed in the data at the time of lever press, but otherwise fits the data poorly (SSE: 49.87; AIC: 21.78). Again, RCH2 gives no reason to expect the pre-stimulus peak in responding. Furthermore, the interference curve is too narrow for the model to account for the difference of response levels observed between conditions (irrespective of *h*2); for the given height (1.84), the breadth would have to be an infeasible eight time periods in order to achieve the observed stimulus-period response levels (SSE: 94.35). This is a general difficulty for RCH; any feasibly sized interference cannot account for the extent of the observed group differences.

One final effort to accommodate response competition is worth considering. If the delay model and the interference model were combined by subtracting an interference curve (as in RCH2) from a model with the position unspecified (as in RCH1), a three-parameter model, a closer fit to the data should be achieved. The resulting model (figure 6, lower panel) is a disjoint collage of RCH1 and RCH2, with the same too-late peak of RCH1 (*p* = 16.64), and a subtraction that fits only the initial nadir (*h*2 = 1.08; *k*2 = 0.13) as in RCH2. The subtracted curves do not appreciably overlap. By hypothesis, the interference curve is expected to interfere with the bump in responding, not several seconds earlier. Combining the models this way produces only a slightly better fit (SSE: 37.00; AIC: 23.08).

Although this may not exhaust all ways of modelling the effects of response competition, the difficulties these attempts raise appear general. The hypothesis does not appear capable of explaining the magnitude or pattern of difference in response levels observed.

#### 4.2.4. Behavioural sequence model

**Figure 7.**
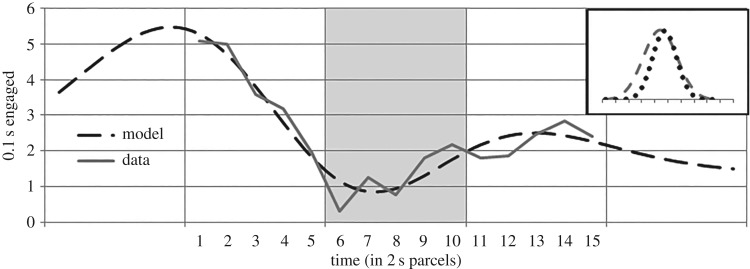
The behavioural sequence model, LD_BS_(*x*), shown with the observed LD condition feeder-directed responding. Vertical lines denote 10 s periods. Shading indicates the stimulus period (6–10). Inset: the focal-appetitive (grey dashed line) and consummatory (dotted) mode models that together produce the behavioural sequence model.

The behavioural sequence model with optimal parameter values is illustrated in figure 7 (focal-appetitive curve: *h* = 10.23; *p* = 4.67; *k* = 5.19; consummatory curve: *h* = 10.13; *k* = 3.70; as above, baseline = 1.38 was given). This model fits the data well (SSE: 2.35; AIC: 10.52). The relationship between the models for focal-appetitive and consummatory modes exhibits several features of Silva *et al*.'s [24–26] diagram: ‘consum’ was later and narrower than ‘focal’ (*p*_consum_ > *p*_focal_; *k*_consum_ < *k*_focal_), was temporally contained by ‘focal’ (*p*_consum_ − *k*_focal_ < *p*_focal_), and reaches above ‘focal’ only briefly, just after ‘focal’ peaks (at *t* = *p*_consum_, *h*_consum_(*t*) > *h*_focal_(*t*)). Even without specifying these as constraints to the parameter search, blind optimization reproduced these relationships drawn in Silva *et al*.'s [24–26] illustrative diagram (apparent in inset of figure 7). Note that lever contact appears in temporally narrower spurts than nosing of the feeder, which in turn is narrower than far platform spurts, as predicted of patterns of consummatory, focal-appetitive, and general-appetitive behaviours, respectively.

Since all but one of the models considered failed to account for the data even qualitatively, there is no quantitative contest, and further quantification of fit is superfluous. Still, the quantitative comparison concurs with the qualitative: AIC of the behavioural sequence model (10.52) is substantially more favourable than those of the presented alternatives (15.89–23.08). Bayesian information criterion of model selection, which carries greater penalties for model complexity, yields the same conclusion (behavioural sequence model: 12.03; alternative models: 16.19–23.99).

#### 4.2.5 Response in the absence of the tone

**Figure 8.**
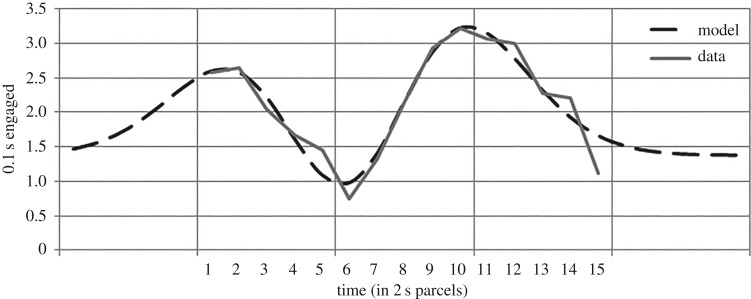
Nosing of the feeder among RI condition subjects before and after moments of lever contact (at time 6). Observed behaviour is shown, along with the best-fitting behavioural sequence model.

Although lever contact by RI subjects produced no stimulus, these rats contacted the lever no less, and their behaviour before and after contact can be analysed as was done for LD subjects. Figure 8 shows the feeder-directed responding of RI subjects before and after moments of lever contact, along with a behavioural sequence model, as described above, with optimal parameters. In this case, we were able to specify not only the position of the consummatory curve (*p*_consum_ = 6), but also its height, which we assumed to be the same as among LD subjects (*h*_consum_ = 10.13), leaving four unspecified parameters. The resultant model fitted the data tightly (SSE: 0.67). In this model, focal(*x*) was lower (*h*_focal_ = 9.75) and later (*p*_focal_ = 6.19) than the corresponding model of LD subjects, and both curves were narrower (*k*_focal_ = 3.37, *k*_consum_ = 2.67). As for LD subjects, we see an anticipatory peak of focal-appetitive behaviour, which falls over the seconds preceding lever contact, leading to a period of below-baseline responding, followed by a second peak. Hence, the pattern expected by a behaviour systems view is observed even in the absence of a self-produced stimulus, including aspects with which the alternatives have difficulty.

## 5. Discussion

Blaisdell *et al*. [1] showed that rats nose a food trough less in response to a self-produced conditional stimulus than to the same stimulus otherwise. We replicated this result. Three explanations of their results have been proposed—causal model theory [1], response competition [6], and here, a hypothesis in terms of motivational sequences, based on behaviour systems theory. These three hypotheses make qualitatively dissimilar predictions which permit their differentiation. The causal model and response competition hypotheses anticipate the observed topography of behaviour in neither form nor composition. The behaviour systems-based hypothesis, by contrast, predicts the observed data well, and in greater specificity of form and breadth of response type.

### 5.1. Anticipation of lever contact

When a rat grabs or bites the lever in a food conditioning chamber, it is indicating that it is in the course of a progression of motivation that began some time before, such that behaviour immediately prior to lever contact will be predictable. The rat about to take the lever in hand or maw is in a very particular motivational condition, which should be visible in its behaviour. In particular, it will have just passed a peak of focal-appetitive behaviour. Elevated nosing of the feeder immediately prior to lever contact was striking (cf. [5], which reported no such differences, but note the differences of measurement and their multiple data transformations, particularly their dropping of zeros). Such an anticipatory peak is an articulated expectation of behaviour systems theory (e.g. [24–26]), and predicted by the behavioural sequence model for any parameter settings that preserve the hypothesized relations among response modes. The model moreover predicts the finding that feeder-directed responding falls for several seconds prior to lever contact (figure 7). These results are awkward for the alternative hypotheses.

### 5.2. Other causal models

Blaisdell *et al*. [1] defended the model diagrammed in figure 1 (see footnote 1). However, this is not the only model consistent with CMT, their training procedures, and their results. In particular, CMT is not a theory of learning, and so it provides little guidance on how specific training procedures relate to acquisition of specific causal models. The assumption that subjects would interpret precedence as causation is more intuitive [30] than principled. It has already been noted [31] that Blaisdell *et al*.'s [1] ‘common-cause’ training procedure would not be expected to produce the hypothesized common cause model by a Bayesian learning model, since the tone was not presented independently of food. The matter is analogous to earlier concerns with using explicitly unpaired conditions as control procedures [32]. The concern is worth acknowledging, but does not indicate violation of the Markov condition, as claimed by critics [31]. By some models of learning, conditional independence in the training data is necessary for the learner to acquire a representation that exhibits conditional independence (e.g. [33]), but not by others (e.g. [34]; discussed in [35]), and CMT is committed to none of them [34].

**Figure 9.**
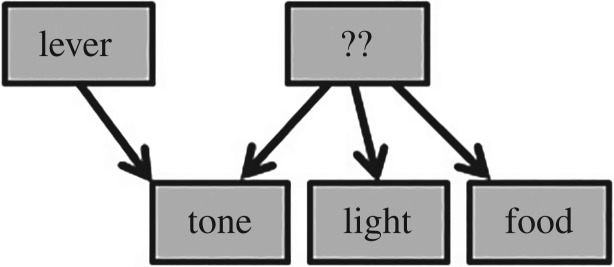
An alternative causal model in which the common cause of tone and food is unseen. This is consistent with the results of Blaisdell *et al*. [1] and Dwyer *et al*. [6], and avoids conceptual problems with the Blaisdell *et al*. [1] model.

One consequence of this flexibility regarding learning is that a number of causal models are consistent with the training in [1]. Among these alternatives, the model diagrammed in figure 9 deserves attention. By figure 9, the light is just another effect of an unseen common cause. This retains the relationships among the tone and food and lever, and so the prediction in focus remains. This model is simpler, better supported empirically, and avoids some of the conceptual foibles of figure 1.

Notably, Blaisdell *et al*. [1] violate an assumption important for the rationale of their study in testing subjects in the absence of the light. By Bayesian networks, two effects of a common cause become dependent only when occurrence of the causing event is unknown. In Blaisdell *et al*.'s [1] situation, however, the purported cause, the light, is not unknown, but absent. Seeing that the light is off, the probability of food appearing should therefore be regarded as independent of the presence of the tone. Hence, given the model in figure 1, both groups should respond similarly. It is an interesting fact that the tone in this study evokes approach to the food trough, but this is not a prediction of the model Blaisdell *et al*. [1] highlight (figure 1), unless one assumes that rats are insensitive to the distinction between absence of knowledge and knowledge of absence, which would appear to undermine normative models of causal reasoning (cf. [36]). The model in figure 9 provides one solution: the common cause of tone and food is unspecified, and so unknown, as required for the prediction in focus.

Figure 9 is simpler because it does not require the ungainly premise that rats will interpret the light as a cause of food or tone just because it precedes it. Empirically, this model fits Dwyer *et al*.'s [6] finding that analogous group differences obtain in simultaneous and sensory preconditioning control conditions, which is another difficulty for Blaisdell *et al*.'s [1] interpretation. Of course, CMT fares no better with figure 9 than with figure 1 in face of the data of the present study. However, insofar as a Bayesian networks analysis is still viable, figure 9 may provide a better starting point than the model the community has been focusing on. Other graphical model-based theories may be clearer about what structure the learner is hypothesized to have acquired.

### 5.3. Control conditions of Blaisdell *et al*. [1]

Blaisdell *et al*. [1] provided two ostensible control procedures, ‘direct-cause’ (i.e. simultaneous conditioning) and ‘causal-chain’ (i.e. sensory preconditioning, cf. [4]), and stressed that response-dependent groups showed reduced responding only in the experimental condition. By contrast, Dwyer *et al*. [6] showed analogous differences with these control procedures as well. This positive finding is much weightier evidence than Blaisdell *et al*.'s [1] mere failure to reject the null hypothesis. Absence of an effect was not properly established, such as by equivalence testing [37], appropriate if weight is to be placed on a negative finding. In either case, however, the interpretation is not clear, for these ostensible control procedures neither control all of the relevant variables nor differentiate among the hypotheses on offer.

The control procedures were designed to discourage explanations in terms of artefacts produced by the response-dependence of the one group's presentations. It was important for Blaisdell *et al*. [1] to show no group differences in the control procedures, not because it was a positive prediction of CMT, but because it would counter explanations such as response competition. By showing that the difference obtains even in these control procedures, Dwyer *et al*. [6] undermined the pretence that those procedures silence the challenge of response competition. The parsimony of response competition makes it a weighty challenge. Addressing this challenge now requires a different strategy.

To be clear, both sets of results are consistent with CMT, as well as with Bayesian networks. Neither theory specifies the appropriate manner of training to acquire particular causal models. Any of the training procedures in [1] could have produced their hypothesized causal-model representation (figure 1), and other causal-model representations also consistent with the three training procedures could produce the same set of results. Figures 1 and 9 show just two of many models that are consistent with CMT, as well as with all training procedures and results of both [1] and [6]. Assertions that the control conditions in [1] are critical for CMT requires one to follow a trail of unarticulated intuitions to the specific model highlighted (figure 1). Alas, CMT is not sufficiently specific to give us just one model for a given training procedure, nor just one way to train a given model.

Furthermore, those control procedures involve multiple moving parts. With the pretence of varying ‘causal models’, the control procedures in [1] leave variables known to affect learning, such as timing and associative strength, uncontrolled. Most obviously, the ‘direct cause’ training procedure (simultaneous conditioning) is more direct than the very indirect training in the experimental condition, and so a given number of pairings is expected to produce much stronger conditioning. In the case of the ‘causal chain’ (sensory preconditioning) condition, the timing of the tone with respect to food delivery is very different from the experimental condition (T–L; L-food versus L–T; L-food). That such variables will change the response topography should not be controversial, and yet proponents (e.g. [38,39]) and critics (e.g. [31]) alike have claimed that the Blaisdell *et al*. [1] demonstration defies alternative theories. These claims were made too hastily.

### 5.4. Evidence counter to the causal model hypothesis

Blaisdell *et al*. [1] reasonably attributed the lower responding to self-produced tones to a reduction in expectation of food. However, it is not the case that responding is generally weaker on lever-produced presentations, as their view predicts. On the contrary, rats responded heartily to self-produced stimuli; though they nosed the feeder less, other feeding-related behaviours were greater on such presentations. As Dwyer *et al*. [6] demonstrated, and we replicated, subjects spent more of their time working the lever on presentations that were self-produced. We furthermore found that movement around the chamber, another response measure that appears among rats in the context of feeding, was also high on such presentations (see also [9]). That certain responses were heightened on self-produced presentations, rather than reduced, clashes with any interpretation based solely on reduction of expectation. This is especially true once the enthusiastic responding on such trials is recognized to be feeding-related. Responding is not weak on self-produced presentations; rather, the response topography differs qualitatively.

By a behaviour systems view, lever contact, and hence the self-produced stimuli, occur not at a low of expectation, but at an apex of feeding motivation, at which the dominant response is consummatory rather than appetitive. However, although consummatory behaviour dominates, even focal-appetitive motivation is expected to be at a high for LD subjects, as is apparent in figure 2. Comparison of the focal-appetitive component of the behavioural sequence model (*h* = 10.23; *k* = 5.19) with the corresponding model of feeder-directed responding in condition RI (figure 4; *h* = 2.18; *k* = 2.59) reveals that the former is much larger, in both height and breadth. This again suggests that feeding-related motivation is greater for the rat who pressed for the tone (LD), than for her yoked partner (RI). The expectation of heightened motivation on lever-induced presentations is in clear conflict with the causal model explanation by which expectation of food will be reduced on self-produced presentations. Note that this similarly speaks against any view that predicts the Blaisdell *et al*. [1] demonstration on the basis of a reduction of expectation, including associative models (e.g. [21]).

### 5.5. Evidence counter to the response competition hypothesis

The response competition explanation [6] relies squarely on the claim that lever contact will interfere with nosing of the feeder in 10 s periods. If LD subjects were slower to respond on account of starting the trial with a lever press, this could produce a difference in overall response levels during the 10 s period measured. At shorter intervals, this might appear obvious, but it is less obvious that having contacted a nearby lever in a small chamber should constitute a measurable impediment to a rat's feeder-directed responding over a period as long as 10 s. One might reasonably expect the opposite, since LD subjects are necessarily always quite close to the food trough at the onset of each presentation, on account of having just grabbed the lever, whereas RI subjects might be anywhere in the chamber. An appropriate solution is to quantify how these behaviours correlate.

Dwyer *et al*. [6] reported a negative correlation of lever contact and nosing the feeder in their experiments, but only for the period of stimulus presentation, and only when tested irrespective of condition. Note that this correlation is another way of expressing the previously noted result that lever contact persists in the lever-press initiated condition while nosing of the feeder is reduced. Dwyer *et al*. [6] found no such correlations within conditions in either experiment, and also not in a post-stimulus period (10–20 s after presentation). This pattern of no correlation in 10 s periods of test sessions, except during the tone, concords with our results. This might have been sufficient to convince a reader of Dwyer *et al*. [6] that response competition does not explain Blaisdell *et al*.'s [1] results.

**Figure 10.**
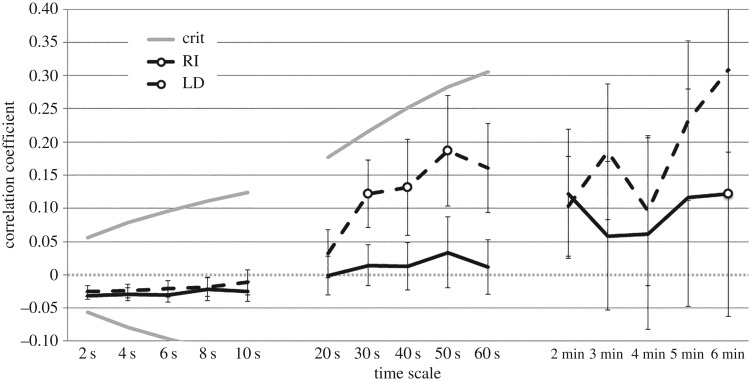
Correlation between lever contact and nosing the feeder for all time scales considered. Grey lines indicate significance criteria for individual correlations. Reliable correlations are indicated by circles (binomial tests, *p* < 0.05). Error bars indicate s.e.m.

A behaviour systems view focuses on how behaviours relate to each other. As expressions of the same system, lever contact and nosing the feeder are expected to occur in sequences, and so to correlate at some time scales. Though this is ostensibly the opposite prediction of response competition, it depends crucially on the scale of measurement. Behaviours that occur in sequences should simultaneously correlate positively at some time scales and negatively at others. Accordingly, we found that correlation coefficients for lever contact and nosing the feeder over test sessions were negative only at the shortest time scales (2, 4, 6 s segments), and only insubstantially (mean *r* = −0.03), while the same data were correlated positively at intermediate time scales (30, 40, 50 s segments), and were uncorrelated at longer time scales (figure 10). Again, there was no correlation between these measures when considered in 10 s segments, as also reported in Dwyer *et al*. [6], contrary to the response competition explanation. Note that our chambers are larger than those used in [1] and [6], potentially affording greater opportunity for responses to interfere with each other.

Note furthermore that the response competition explanation requires more than a merely significant negative correlation between lever contact and nosing the feeder, but a substantial one. Attempting to explain an effect of one variable in terms of covariation with another requires establishing that the association is sufficiently strong to account for the observed effect. A correlation unable to account for the whole effect loses any ostensible parsimony. In the present case, the degree of association between group membership and nosing of the food trough during the CS was substantial, approximated as *η*^2^ = 0.67 (from *F* and d.f., perhaps charitably [40]). In order to account for these data solely in terms of association with lever contact, therefore, would require a strong correlation (beyond −0.8, according to these estimates).

If lever contact impedes nosing of a feeder within nose-length, one might expect it to be an even greater impediment to being at the far end of the chamber. Contrary to this expectation, lever-pressing subjects managed to be at the far end of the chamber at least as much as RI subjects during the tone. This is hard to reconcile with appeal to response competition as an explanation for less nosing of the feeder during the same period.

What does it mean that rats persist with lever contact? Lever contact is not distributed evenly over time, but occurs in spurts and cycles. That there is greater lever contact on lever-produced presentations comes as no surprise; this finding does not contribute to a response competition explanation, since it is given by the procedure that LD subjects will have just pressed at each presentation. A response competition-based hypothesis needs nothing more, and gives nothing more in terms of specific predictions of patterns of lever contact.

### 5.6. Interdependence of motivational modes is not response competition

A behaviour systems view attributes the pattern in focus to a manner of competition among motivational modes, such that related behaviours occur in meaningful sequences rather than all at once. Despite superficial similarity with the response competition explanation, an important distinction is worth stressing. In some cases, a conflict among behaviours may be an artefact of a specific apparatus or procedure. For instance, increasing the spacing between two response levers is likely to affect performance without reflecting a difference in competence of the animal. In some cases, however, dependence among behaviours is a quality of the actor, and contributes to apt timing of behaviour in meaningful ways. A foraging rat, for instance, might advantageously alternate between exploiting a known food source and exploring abroad. The same rat might interleave grooming or defecating with only some sorts of foraging, court before mating, not mate while hunting, not hunt while sleeping, and generally perform behaviours in sequences that may be complexly determined but far from random. Many cases of separation among behaviours are not produced by simple physical incompatibilities, but by the structure of motivational systems. We present evidence that Blaisdell *et al*.'s [1] demonstration is such a case.

An important difference among these views concerns the applicability of normative or rational theories, such as Bayesian networks. Only if a pattern of behaviour reflects a competence of the animal might application of normative theories, in principle, be appropriate. This is true whether the competence involves figuring with mental models or well-structured motivational systems.

## 6. Conclusion

Blaisdell *et al*. [1] showed that responding to a conditional stimulus was less when it was self-produced than otherwise, in a way that matches a view of causal reasoning. We analysed this demonstration, considering the specific apparatus, procedures and measurement scheme used, with attention to the motivational system evoked, and the way the structure of that system constrains and directs behaviour. Just using data from a partial replication of the original experiment, there are several lines of evidence that bear on the questions raised.

First, our subjects did show reduced feeder-directed responding to the self-produced stimulus relative to response-independent, reaffirming the key prediction. Second, we show that prior attempts to explain this in terms of causal-model representational maps, or to dismiss it as response competition, fail to account for a closer look at the data, quantitatively and even qualitatively. Much of our critique regards omissions that become apparent with a change in perspective, but there are also patterns of results that are directly counter to both views. This failure extends more generally to views that explain the effect in terms of reduction of expectation (e.g. [21]) or processing limitations [22]. Subjects are neither discounting self-produced stimuli, nor are they distracted by the lever press itself.

Our behaviour systems-based hypothesis does account for the observed pattern of results, and makes sense of a wider range of behaviour. By presenting the stimulus only at the moment of lever contact, the experiment focuses on a very specific motivational circumstance. When lever contact is viewed, not as an arbitrary operant, but as a part of a sequence of behaviour related to feeding, the observed response topographies become interpretable.

An important aspect of CMT is that it conflates two different kinds of question. There are questions about rationality, and questions about mentality. We have no flags to wave against either kind of analysis, in principle. However, the data of the present and related studies are relevant to rationality, and they have been used to draw conclusions about mental manifestation. An analysis in terms of rationality leaves gaps, and how those gaps are filled warrants care.

What does this say about causal reasoning in rats? In principle, Bayesian networks still provides a permissible description of our subjects' behaviour. However, this alone says very little. Drawing a connection between this very abstract description and an understanding of rats' behaviour or cognition requires theoretical bridgework. CMT provided one such answer, but one that our data oblige us to reject. Furthermore, even if there were clarity about how to apply these hypothetical network models to cognition in the animal, the Bayesian networks analysis only makes predictions if we specify a structure. Doing so in this case required commitment to a complex set of further assumptions, which, whether properly attributable to CMT or not, we found insufficiently justified. The response competition explanation was important to address in this regard, for it suggested the demonstration may have been a consequence of the apparatus or procedure, rather than a quality of the animal. Rejection of this hypothesis, therefore, but not affirmation of CMT, is necessary for analysis of the experiment in terms of Bayesian networks to be appropriate. Our rejection of both the causal model and response competition hypotheses, therefore, leaves Bayesian networks unscathed, but ineffectual. Affirmation of a single, isolated prediction, heavily loaded with weakly justified assumptions is only very weak support. Even so, insights from graphical models-based perspectives are worth retaining. Although still largely human-centred, theoretical developments among views that reference graphical models are maturing in promising directions (e.g. [33,41–43]; see also [3,44]; for an overview of specifically non-human causal reasoning work, see [45]).

More positively, the present study supports an integrative approach. The framing graphical model-based views, like Bayesian networks, require may be provided by behaviour systems, such that an analysis of the focal demonstration in terms of causal reasoning may yet be appropriate. Behaviour systems theory is not a theory of causal reasoning, but it can provide theoretical scaffolding for such a theory, to make sense of a wider range of behaviour. Having an analysis of the experimental situation in terms of motivational systems is meaningful on its own, but this gains additional significance in integration with a complementary analysis in terms of cognitive processes or rationality. A fuller description of the behaviour of rats in these circumstances reveals striking behavioural patterns not foreseen by theories of causal reasoning (see also [9]). If the experiment meaningfully captures causal reasoning, these variables may have bearing on how rats deal with cause and effect in analogous circumstances.

Finding against a specific theory of causal reasoning does not entitle denial of rats' capacity for causal reasoning. Even if relevant theories were sophisticated and plentiful, and none of them stood to empirical testing, the conclusion that rats are incapable of causal reasoning would still not follow. If theories of gravity all fail, the phenomenon would be no less present than before, just like the ability of rats to deal well with cause and effect. The cognitive sophistication of rats does not turn on our success or failure to describe it.

## Footnotes

1 There is ambiguity regarding whether the lever–tone association is conceived as part of the subjects' representational model, or should be represented differently, as ‘intervention’. The model as drawn is more general than versions that represent intervention differently. Such distinctions affect neither Blaisdell *et al*.'s [1] analysis nor the present analysis. The status of intervention among rats is a separable, remaining question, not to draw assumptions about lightly [9].

2 Note that lever contact, for instance, is neither exclusively nor essentially a consummatory feeding behaviour. Rather, the lever affords a particular manner of expression for a rat taken by consummatory motivation in the specific context of the experiment (cf. [27]). Some earlier writing (e.g. [24–26]) has favoured the phrase ‘handling/consuming’ as a less abstract alternative to ‘consummation’. Despite superficial similarity between these words, consume and consummate are distinct not only in contemporary usage, but also in their Latin origins: *consūmere* (to deplete, exhaust), and *cōnsummāre* (to complete, accomplish), respectively. The bites of a rat engaged in consummatory lever contact are conceived as the latter (after [23]).

## Data accessibility

All reported data can be accessed from the Dryad Digital Repository [46]: http://dx.doi.org/10.5061/dryad.20h4r.
